# Regional Cerebrospinal Fluid Motility as a Key Determinant of Soluble Amyloid-β Levels and Kinetics

**DOI:** 10.21203/rs.3.rs-10530209/v1

**Published:** 2026-08-03

**Authors:** Helia Hosseini, Aristeidis Sotiras, Chihiro Sato, Donald L. Elbert, Braden Yang, Nelly Joseph-Mathurin, Vitaliy Ovod, Bruce W. Patterson, Brian A. Gordon, Sarah J. Keefe, Suzanne E. Schindler, Tammie L. S. Benzinger, Arash Nazeri

**Affiliations:** 1 Mallinckrodt Institute of Radiology, Washington University School of Medicine, St. Louis, MO, United States; 2 Institute for Informatics, Data Science & Biostatistics, Washington University School of Medicine, St. Louis, MO; 3 Department of Radiology, Perelman School of Medicine, University of Pennsylvania, Philadelphia, PA; 4 Center for AI and Data Science for Integrated Diagnostics (AI2D), Perelman School of Medicine, University of Pennsylvania, Philadelphia, PA, United States; 5 Department of Neurology, Washington University School of Medicine, St. Louis, MO, United States; 6 Hope Center for Neurological Disorders, Washington University School of Medicine, St. Louis, MO; 7 Tracy Family SILQ Center, Washington University School of Medicine, St. Louis, MO; 8 Department of Neurology, University of Washington, Seattle, WA, United States; 9 Center for Human Nutrition, Washington University School of Medicine, St. Louis, MO, United States; 10 Department of Biomedical Engineering, Washington University in St. Louis, St. Louis, MO, United States

**Keywords:** CSF flow, low b-dMRI, amyloid β clearance, glymphatic system, intramural peri-arterial drainage

## Abstract

Alzheimer’s disease and cerebral amyloid angiopathy are characterized by the accumulation of amyloid-β (Aβ) species, yet the human fluid-physiological mechanisms that regulate soluble Aβ transport and clearance remain poorly understood. Aβ40, the dominant vascular Aβ species, remains relatively soluble and accumulates preferentially along vascular basement membranes, making it especially relevant to CSF-mediated clearance pathways. Here, we used non-invasive low b-value diffusion MRI to quantify regional effective CSF motility in a large multimodal cohort from the Knight Alzheimer Disease Research Center, including participants with CSF biomarkers (n = 502), plasma biomarkers (n = 459), amyloid PET imaging (n = 270), and stable isotope labeling kinetics data (n = 40). Effective CSF motility was quantified as mean pseudo-diffusivity (MΨ) and mapped using CSF pseudo-diffusion spatial statistics with a data-driven CSF Waterways atlas. Higher regional CSF motility was consistently associated with higher CSF Aβ40 concentrations, with convergent voxel-wise and region-of-interest effects across ventricular and extra-axial CSF compartments that were most prominent in peri-Sylvian CSF pathways. Across regions, CSF motility measures collectively explained approximately 18% of the variance in CSF Aβ40. A weaker, spatially restricted association was also observed with plasma Aβ40. In contrast, associations with CSF Aβ42 were smaller, anatomically limited, and further attenuated among participants with amyloid deposition on PET. In participants with stable isotope labeling kinetics, higher transventricular and cranio-cervical CSF motility was associated with earlier peak enrichment of labeled Aβ40 and Aβ42, while greater lateral ventricular motility was associated with faster fractional turnover of both peptides. Collectively, these findings identify effective CSF motility as an important physiological determinant of soluble Aβ levels in plasma and CSF, linking regional CSF dynamics to in vivo Aβ transport and turnover, with potential relevance to cerebral amyloid angiopathy and Alzheimer’s disease.

## 1. Introduction:

The pathogenesis of Alzheimer’s disease (AD) is closely linked to the progressive accumulation of pathologic amyloid-β (Aβ) species^1,2^. Cleavage of the amyloid precursor protein gives rise to multiple Aβ peptide isoforms, of which Aβ42 and Aβ40 are the most pathologically relevant. Although closely related, these isoforms differ markedly in their physical properties and aggregation patterns^3^. Aβ42 is more prone to misfolding and aggregation and represents the predominant component of parenchymal amyloid plaques in AD^4^. By contrast, Aβ40 is more commonly associated with cortical and leptomeningeal vascular amyloid deposits characteristic of cerebral amyloid angiopathy (CAA)^5,6^.

The removal of Aβ peptides from the brain involves multiple complementary pathways, including proteolytic degradation, transport across the blood–brain barrier, and cerebrospinal fluid (CSF)-based clearance^7–9^. As alternative pathways diminish with aging, CSF-based clearance assumes a proportionally greater role, despite being itself vulnerable to age-related impairment^7^. Within this clearance pathway, CSF from the subarachnoid space enters the brain parenchyma through the perivascular spaces, driven by a convective flow. Within the glymphatic system framework, CSF entering along perivascular routes exchanges with interstitial fluid (ISF), thereby facilitating clearance of metabolic waste such as Aβ via perivenous efflux pathways^8,9^. In contrast, the intramural periarterial drainage (IPAD) model posits that Aβ is cleared via the arterial vessel walls, cleared along arterial vessel walls, driven by vasomotor oscillations^10–12^. Disruption of these pathways can compromise Aβ clearance, thereby promoting its accumulation within the brain tissue and vasculature^13,14^.

Despite experimental models implicating CSF circulation in Aβ clearance, it is not yet clear whether regional CSF motility in humans relates to soluble Aβ species or cerebral amyloid deposition^13^. Prior contrast-enhanced magnetic resonance imaging (MRI) studies have demonstrated age-related impairments in CSF-based clearance^15^; however, intrathecal contrast administration is invasive, precluding large cohort studies. Therefore, non-invasive methods capable of fast and scalable quantification of regional CSF flow are essential. Low b-value diffusion-weighted MRI (low-b dMRI) addresses this need by sensitizing the signal to the effective motility of CSF across a wide range of flow behavior^16–19^. Using pulsed-gradient spin-echo and fast rapid echo-planar imaging (EPI) readout, low-b dMRI quantifies signal attenuation from pseudorandom CSF displacements to compute mean pseudodiffusivity (MΨ), a metric of effective CSF motility^20,21^. Low-b dMRI has been used to evaluate alterations in CSF effective motility in aging^19,22^, Parkinson’s disease^23^, and normal pressure hydrocephalus^24^.

Importantly, most human studies rely on static Aβ concentrations and amyloid deposition, which do not directly capture underlying transport and clearance dynamics. Stable isotope labeling kinetics (SILK) uses infusion of a nonradioactive labeled amino acid (e.g., ^13^C_6_-leucine) and quantifies tracer incorporation into Aβ over time to derive *in vivo* kinetic parameters reflecting Aβ transport and turnover^25–28^. which directly quantifies Aβ production and clearance kinetics using stable isotope labeling. SILK offers a unique window into Aβ transport processes that are not accessible with amyloid PET or conventional CSF biomarkers, which primarily reflect cumulative deposition or steady-state levels.

**Figure 1.**
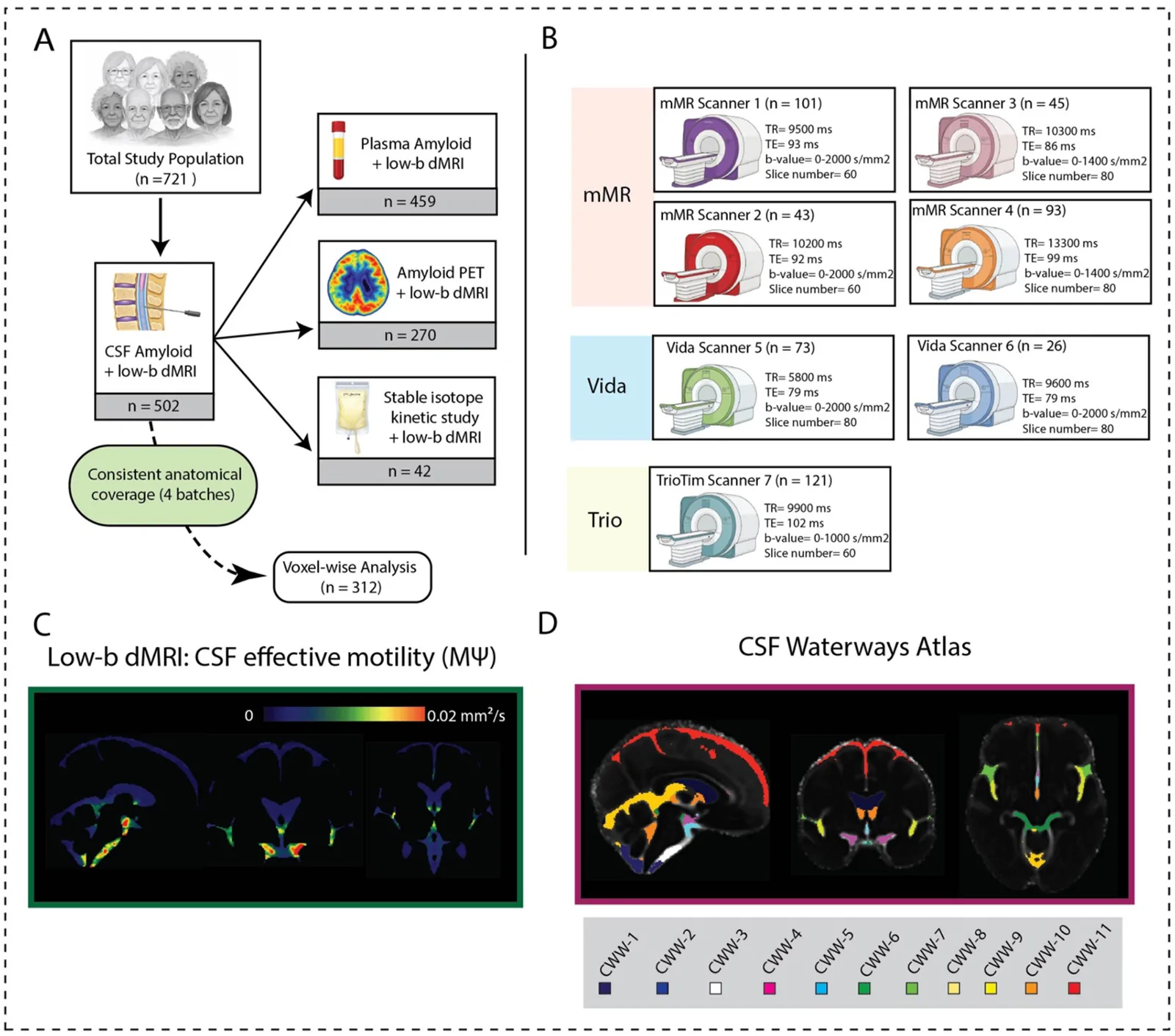
Overview of the study design and MRI acquisition across scanner batches. **(A)** Overview of the study workflow and sample stratification based on data availability **(B)** The seven MRI acquisition batches used in the study with different imaging parameters. **(C)** Representative sagittal, coronal, and axial views demonstrating spatial distribution of CSF motility (MΨ) derived from low-b diffusion MRI. Warmer colors indicate higher MΨ (scale: 0–0.02 mm^2^/s). **(D)** The CSF Waterways atlas: CWW-1, lateral ventricles; CWW-2, cranio-cervical junction; CWW-3, superior premedullary cistern; CWW-4, chiasmatic cistern; CWW-5, prepontine cistern; CWW-6, perimesencephalic cisterns; CWW-7, Sylvian fissure (opercular segment); CWW-8, Sylvian fissure (cisternal segment); CWW-9, supracerebellar and velum interpositum cisterns; CWW-10, third and fourth ventricles and CWW-11, extra-axial CSF spaces.

We hypothesized that regional alterations in CSF circulation influence amyloid clearance from the brain. To test this, we leveraged a large multimodal dataset encompassing low-b dMRI, amyloid positron emission tomography (PET), and biofluid Aβ measures (n = 502; Figure 1). Using CSF pseudodiffusion spatial statistics (CΨSS) together with the CSF Waterways (CWWs) atlas^21^, we evaluated how regional effective CSF motility (MΨ) relates to Aβ concentrations in CSF and plasma, as well as to brain amyloid deposition. In a subset of participants, we additionally examined associations with in vivo Aβ turnover dynamics measured using SILK. Greater CSF motility was associated with higher CSF and plasma Aβ40 concentrations, whereas associations with Aβ42 were weaker and largely confined to individuals with negative amyloid PET scans. Nonetheless, no cross-sectional relationship was observed between CSF motility and parenchymal amyloid deposition on PET. SILK analyses further demonstrated that regional CSF motility was associated with earlier peak tracer enrichment and faster turnover kinetics, supporting a role for CSF dynamics in modulating Aβ transport and clearance.

## 2. Methods

### 2.1. Participants

This study utilized imaging data from the Knight Alzheimer Research Imaging (KARI) dataset and corresponding biofluid data collected through the Washington University Knight Alzheimer Disease Research Center (Knight ADRC). Participants aged 50 years or older were included from ongoing memory and aging studies corresponding to the 23^rd^ and 26^th^ data freeze. All procedures were reviewed and approved by the Institutional Review Board of Washington University School of Medicine in St. Louis, and written informed consent was obtained from all participants or their legally authorized representatives. A subset of participants additionally had available Aβ SILK tracer measurements. The SILK studies were performed at Washington University in St. Louis and were approved by the Washington University Human Studies Committee and the General Clinical Research Center Advisory Committee.

### 2.2. MRI imaging

Low-b dMRI was performed on multiple 3T Siemens MRI systems (TrioTim, Biograph mMR, and Magnetom Vida) at the Center for Clinical Imaging Research (CCIR), Mallinckrodt Institute of Radiology, Washington University in St. Louis between August 2007 and December 2021. All data were acquired using 2D echo-planar imaging (EPI) with isotropic 2 × 2 × 2 mm^3^ voxels. Across all scanners and protocols, each diffusion gradient direction was paired with a unique b-value. Additional acquisition parameters are provided in Supplementary Table S1.

### 2.3. Low-b dMRI preprocessing and CSF pseudo-diffusion spatial statistics (CΨSS)

At low b-values, the observed pseudo-diffusion tensor **Ψ** reflects two principal contributions: (1) the diffusion tensor **D**, which reflects a combination of random (disordered) motion and intrinsic diffusivity, and (2) the covariance matrix of the 3D velocity probability distribution **V**, which captures linear (ordered) motion. Therefore, **Ψ** can be expressed as the sum of **D** and **V** with τ_d_ denoting the diffusion time^16,17^:

$$\Psi \approx \frac{\tau_{d}}{2}\text{V}+\text{D}$$

Provided that the intrinsic physical properties of CSF (e.g., temperature and viscosity) remain unchanged, the MΨ derived from the **Ψ** tensor serves as an indicator of the variability in flow velocity within a voxel, driven by laminar and/or mixing movements of CSF. To derive MΨ maps from the diffusion-weighted data and perform group-level analyses of CSF flow changes, we applied a preprocessing and CΨSS postprocessing workflow, as described below (Supplementary Figure S1).

Briefly, to correct for susceptibility-induced distortions eddy current-induced distortions and subject motion were corrected using FSL’s *eddy_correct*. The resulting eddy corrected image was then skull-stripped using the brain extraction tool (BET) to generate a brain mask^29^. The multi-b-value diffusion MRI dataset was then divided into two subsets. The low b-value subset (b= 0–450 s/mm^2^; sampled in ~50 s/mm^2^ increments) was used to fit a diffusion tensor model for calculating the mean MΨ maps, which reflect pseudorandom CSF flow. The high b-value subset (b≥ 500 s/mm^2^) and b0 images were used to fit a separate diffusion tensor model to generate fractional anisotropy maps. The resulting low-b mean diffusivity (MΨ) and high-b-value fractional anisotropy maps were then fed into the Atropos^30^ to derive partial volume estimates of CSF and white matter using a two-tissue model. Similar to gray matter-based spatial statistics^31,32^, partial volume estimates were combined to synthesize pseudo-T1-weighted images, using gray matter, white matter, and CSF probability maps all derived from diffusion data. MΨ maps were thresholded to exclude physiologically implausible values. Batch-specific templates were then constructed using both MΨ and pseudo-T1-weighted images as inputs to the *antsMultivariateTemplateConstruction2.sh* workflow^33^. CSF probability maps were subsequently registered to the common template space. To minimize partial volume effects, the final CSF mask was created by retaining only those voxels where the CSF fraction exceeded 0.8 in at least 75% of the subjects. Voxels with CSF fraction less than 0.8 were then filled with the average of the surrounding satisfactory CSF voxels. Finally, spatial smoothing was then applied within the resulting CSF mask using the AFNI’s *3dBlurInMask* function^34^, with a full width at half maximum value of 2 mm.

### 2.4. Amyloid PET imaging

Amyloid PET scans were acquired using [18F] Florbetapir (FBP) or [11C] Pittsburg Compound-B (PiB) and processed using the PET Unified Pipeline (PUP)^35,36^. PET images were processed using an MRI-free pipeline in which scans were spatially normalized to tracer specific templates and spatially smoothed. Standardized uptake value ratios (SUVRs) were computed for global cortical composite region using the whole cerebellum as the reference region. Global amyloid burden was summarized as mean cortical SUVR and, to enable cross-tracer comparability^37^, additionally expressed in Centiloid units derived from the same cortical composite^38^. Details on amyloid PET acquisition and processing protocols are provided elsewhere^37,39^.

### 2.5. CSF and plasma biomarkers

Fasted CSF concentrations of Aβ40 and Aβ42 were measured using the fully automated LUMIPULSE G1200 chemiluminescent enzyme immunoassay platform (Fujirebio, Malvern, PA), following standardized collection and processing protocols. Formal lot bridging was performed to harmonize the full dataset. Full assay procedures and validation details are available in prior publications^37,40–43^. Fasted plasma Aβ40 and Aβ42 concentration were quantified by C2N Diagnostics using an immunoprecipitation-mass spectrometry assay as part of the *Knight ADRC fasted plasma PrecivityAD* dataset. Detailed assay procedures have been described previously^44^.

### 2.6. Stable Isotope Labeling Kinetics

Aβ stable isotope labeling kinetics (SILK)^25^ measures were available for a subset of participants with tracer kinetic data (n=42). Participants received an intravenous infusion of [^13^C_6_]-leucine, with serial hourly CSF samples collected for up to 36 hours during and following tracer infusion via lumbar catheterization under standardized collection protocols^26,28^. Incorporation of the tracer into soluble CSF Aβ40, and Aβ42 was quantified longitudinally using immunoprecipitation followed by mass spectrometry. Kinetic parameters were estimated using a previously validated compartmental modeling framework. From these analyses, we used the apparent amyloid precursor protein production rate (k_APP_), the fractional turnover rate (FTR), reflecting irreversible loss of soluble Aβ peptides; and the time to peak tracer enrichment (peak time), estimated from the fitted tracer enrichment curves. Participants with kinetic parameter values exceeding ±3 z-scores were excluded as outliers (n = 2). Detailed tracer infusion protocols, analytical procedures, and kinetic modeling methods have been described previously^27,28,45^.

### 2.7. CWW region-of-interest analyses

To investigate associations between regional CSF flow alterations and CSF biomarkers, we performed region-of-interest (ROI) analysis using the CSF Waterway (CWW) atlas— a data-driven atlas based on the covariance structure of intracranial CSF flow^21^, previously created using non-negative matrix factorization^46^. The CWW atlas encompasses the following cisternal and ventricular CSF regions: CWW-1, lateral ventricles; CWW-2, cranio-cervical junction; CWW-3, superior premedullary cistern; CWW-4, chiasmatic cistern; CWW-5, prepontine cistern; CWW-6, perimesencephalic cisterns; CWW-7, Sylvian fissure (opercular segment); CWW-8, Sylvian fissure (cisternal segment); CWW-9, supracerebellar and velum interpositum cisterns; CWW-10, third and fourth ventricles; and CWW-11, extra-axial CSF spaces (Figure 1D). The CWW atlas was nonlinearly registered to our study-specific template Using ANTs^33,47^, and mean MΨ values from each CWW region were then extracted with FSL’s *fslstats* tool.

### 2.8. ROI-level harmonization and cross-batch analyses

Associations between CSF MΨ and CSF biomarker concentrations (Aβ40, Aβ42, and Aβ42/Aβ40 ratio) were examined within each CWW region using general linear models adjusted for age, sex. Analyses were conducted separately within each of the seven scanner and imaging acquisition batches. All other region-of-interest statistical analyses were performed following ComBat harmonization between the batches. ComBat harmonization was applied to the mean CSF MΨ values extracted from each of the eleven CWW regions. Harmonization was performed using the *neuroHarmonize* package, modeling scanner batch as the harmonization factor while retaining age and sex as covariates^48–50^. This procedure removed scanner-related effects while preserving biological variance. The resulting harmonized dataset containing CWW-specific CSF MΨ values was subsequently used for association analyses with multiple Aβ biomarkers, including CSF Aβ40 and Aβ42 concentrations, the Aβ42/Aβ40 ratio, plasma Aβ40 and Aβ42 concentrations, and amyloid PET (Centiloid values). Prior to statistical analyses, regional CWW MΨ values were winsorized at ±3 SD within each region to limit the influence of extreme observations. For each Aβ biomarker, linear regression models were fit for each CWW region to assess their link with harmonized CSF MΨ values, adjusting for age and sex.


$$\text{A}\beta \;\text{Biomarker}\sim \text{age}+\text{sex}+\text{CW}{\text{W}}_{\left[\text{Harmonized}\;\text{M}\Psi \right]}$$

Additionally, forward stepwise selection was applied to identify CWW MΨ predictors of CSF Aβ biomarkers’ levels while controlling for age, sex, and brain parenchymal fraction (BPF). At each iteration, the candidate variable that produced the greatest reduction in Akaike Information Criterion (AIC) was added, provided the improvement exceeded 2 AIC units^51^. Significance was assessed at an p_FDR_ < 0.05 across CWW regions to account for multiple comparisons. Given the smaller SILK sample size, nominal two-sided *p* values are reported without correction for multiple comparisons; these analyses should be considered exploratory and hypothesis-generating.

### 2.9. Multi-batch voxel-wise analysis

Since imaging data were acquired across seven distinct scanner batches with varying acquisition parameters (Supplementary Table S1), voxel-wise harmonization was performed to address inter-batch variability while preserving biologically meaningful variance, with age and sex retained as covariates. To do so, batch-specific templates previously generated for each acquisition group were first constructed into a single “template of templates” using ANTs (*antsMultivariateTemplateConstruction2.sh*)^33^ to establish a unified reference space. For this step, only four batches that provided complete brain coverage with 80 slices were included. CSF probability maps from all batches were then nonlinearly registered to this common template. Voxel-wise harmonization of CSF MΨ values in the final CSF mask was subsequently performed using the ComBat algorithm implemented in the *neuroHarmonize* package^48–50^. After harmonization, 312 participants were available for the final voxel-wise analyses of associations between the harmonized CSF MΨ and CSF biomarkers. These association were examined using FSL’s *randomise tool* with 5,000 permutations and threshold-free cluster enhancement (TFCE), with statistical significance defined at family-wise error (FWE)–corrected level of *p* < 0.05. All models were adjusted for age and sex^52^.

## 3. Results

### 3.1. Demographic and clinical characteristics of the imaging batches

For the ROI-based analysis, participants with both CSF biomarker and MRI data acquired within one year of each other were included (n = 502). For the voxel-wise analysis, both CSF biomarker and MRI data within a one-year interval were once again required; however, for this step only four batches that ensured consistent anatomical coverage across the whole brain were included (n = 312). For the additional ROI analyses, participants with MRI and plasma biomarkers (n = 459) and amyloid PET (n = 270) data acquired within one year were included. Among the 502 participants with available CSF biomarkers, the mean age was 69.2 ± 8.4 years, and 275 (54.6%) were female. The majority of participants were cognitively unimpaired (Clinical Dementia Rating [CDR] score = 0; 85.8%). Amyloid positivity was defined using a Centiloid threshold > 18.41^53^, classifying 17% of participants as amyloid positive. Summary descriptive statistics for each batch are presented in Table 1 and Figure 1, and biomarker values are reported in Supplementary Table S2.

### 3.2. Widespread positive associations between CSF motility and CSF Aβ40, with weaker associations for Aβ42

**Figure 2.**
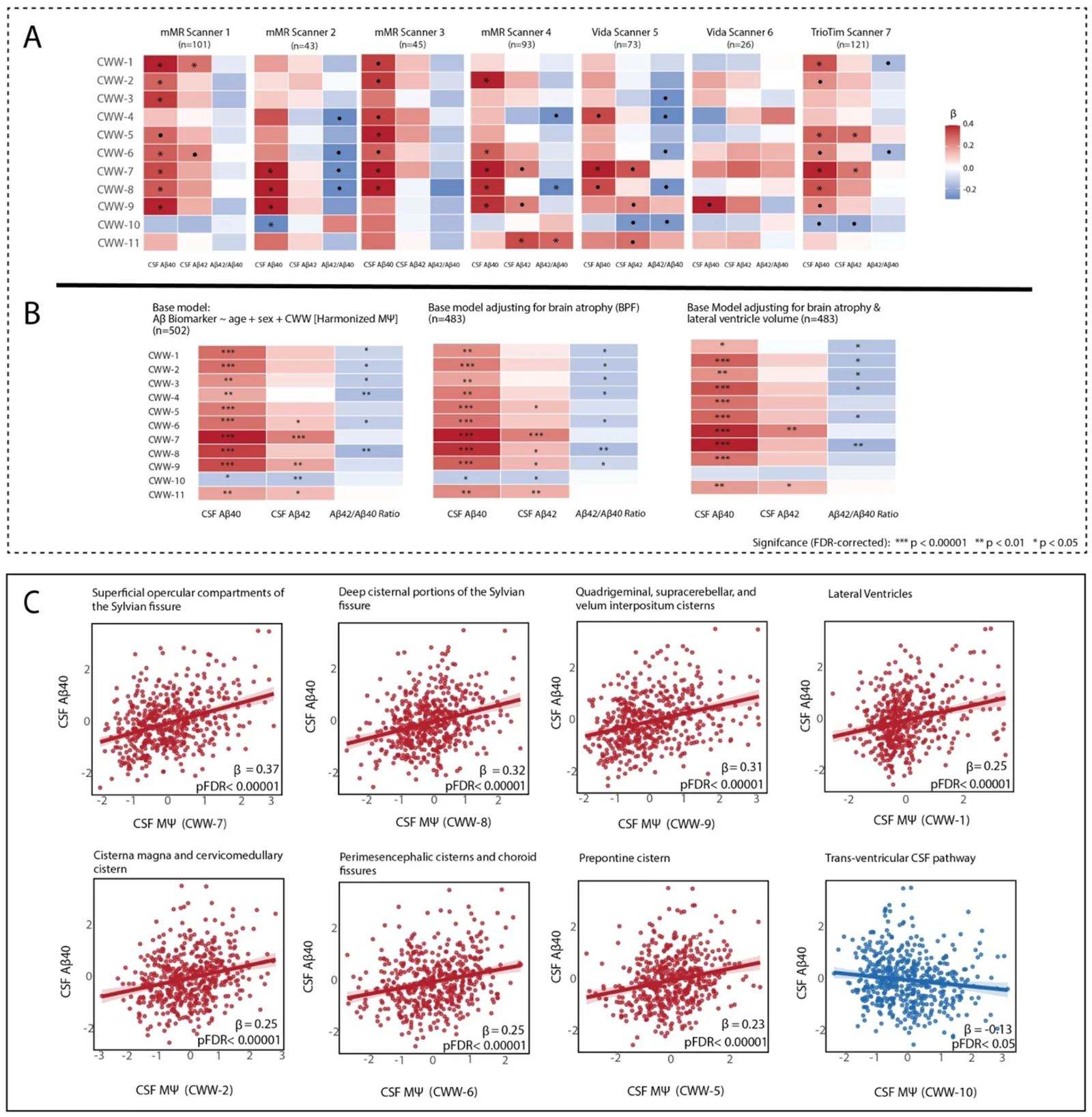
Effects of CSF mean pseudo-diffusivity on CSF Aβ biomarkers across CSF Waterways. **(A)** ROI analyses performed separately within each MRI acquisition batch using the CWW atlas. Association between CSF motility (MΨ) and CSF amyloid-β concentrations (Aβ40, Aβ42, and the Aβ42/ Aβ40 ratio) were examined within each CWW region using the base linear model: CSF Aβ biomarker ~ age + sex + CWW MΨ. Asterisks (*) indicate FDR-corrected significant associations (p_FDR_ < 0.05), and dots indicate nominal significance (uncorrected p<0.05). **(B)** ROI analyses across all batches (n=502) were performed using harmonized CSF MΨ values. Effects of CSF MΨ on CSF amyloid-β concentrations (Aβ40, Aβ42, and the Aβ42/Aβ40 ratio) were estimated using the base linear model: CSF Aβ biomarker ~ age + sex + CWW [harmonized MΨ]. The base model was additionally adjusted for brain atrophy (BPF), and subsequently for both BPF and lateral ventricle volume. Asterisks indicate significant effects after FDR correction: *p* < 0.05 (*), p < 0.01 (**), and p < 0.00001 (***). **(C)** Scatter plots illustrate the associations between harmonized CSF MΨ and CSF Aβ40 concentrations across selected CWW regions in ROI analyses across all batches (n=502), based on the linear base model adjusted for age and sex. Abbreviations: MΨ, mean pseudo-diffusivity; CWWs, CSF waterways; ROI, region-of-interest; BPF, brain parenchymal fraction; FDR, false discovery rate

Across all batches, CWW MΨ showed widespread positive associations with CSF Aβ40 (p_FDR_ < 0.05), accounting of age, sex. Associations with CSF Aβ42 were weaker and less consistent (Figure 2A). Following batch-specific analyses, we performed a multi-batch analysis using harmonized CSF MΨ values and CSF Aβ biomarkers in a total sample of 502 participants. Consistent with the batch-level results, CSF MΨ showed robust positive associations with CSF Aβ40 across all CWWs when accounted for age and sex. The strongest effects localized to CWW-7 and 8 (the Sylvian fissure regions), and CWW-9 (supracerebellar and velum interpositum cisterns) where higher CSF MΨ corresponded to elevated Aβ40 (CWW-7: β = 0.37; CWW-8: β = 0.32; CWW-9: β = 0.31, p_FDR_ < 1 × 10^−6^; Figure 2B and 2C). Given that brain atrophy and ventricular enlargement may affect CSF flow characteristics and CSF biomarker concentrations^21,54^, potentially confounding associations between CSF MΨ and Aβ levels, we repeated the analyses with additional adjustment for brain atrophy (BPF) and lateral ventricular volume. The overall pattern and strength of associations remained largely consistent (Figure 2B). To identify which CWWs explain unique variance in CSF Aβ40 when considered jointly, we performed forward stepwise regression controlling for age, sex, and BPF. CSF MΨ within four regions, CWW-7 (Sylvian fissure, opercular segment), CWW-2 (cranio-cervical junction), CWW-1 (lateral ventricles), and transventricular CWW-10 collectively accounted for 18% of the variance in CSF Aβ40 levels. Consistent with the univariate results, most showed positive associations, with the exception of CWW-10, which demonstrated a significant negative association (p_FDR_ < 0.05).

By contrast, associations with CSF Aβ42 were markedly weaker, with significant effects restricted to the Sylvian fissure (CWW-7: β = 0.2; p_FDR_ = 3.68 × 10^−5^) and supracerebellar and velum interpositum cisterns (CWW-9: β = 0.15; p_FDR_ = 1.7 × 10^−3^) when adjusted for age and sex. Across all CWW regions, the CSF Aβ42/Aβ40 ratio showed a weak but consistent inverse relationship with CSF MΨ. After FDR correction, significant but modest negative associations were identified in multiple basilar cisterns (p_FDR_ < 0.05; Figure 2B), with the strongest effect observed in CWW-8 (β =−0.15, p_FDR_ = 2.7× 10^−3^).

**Figure 3.**
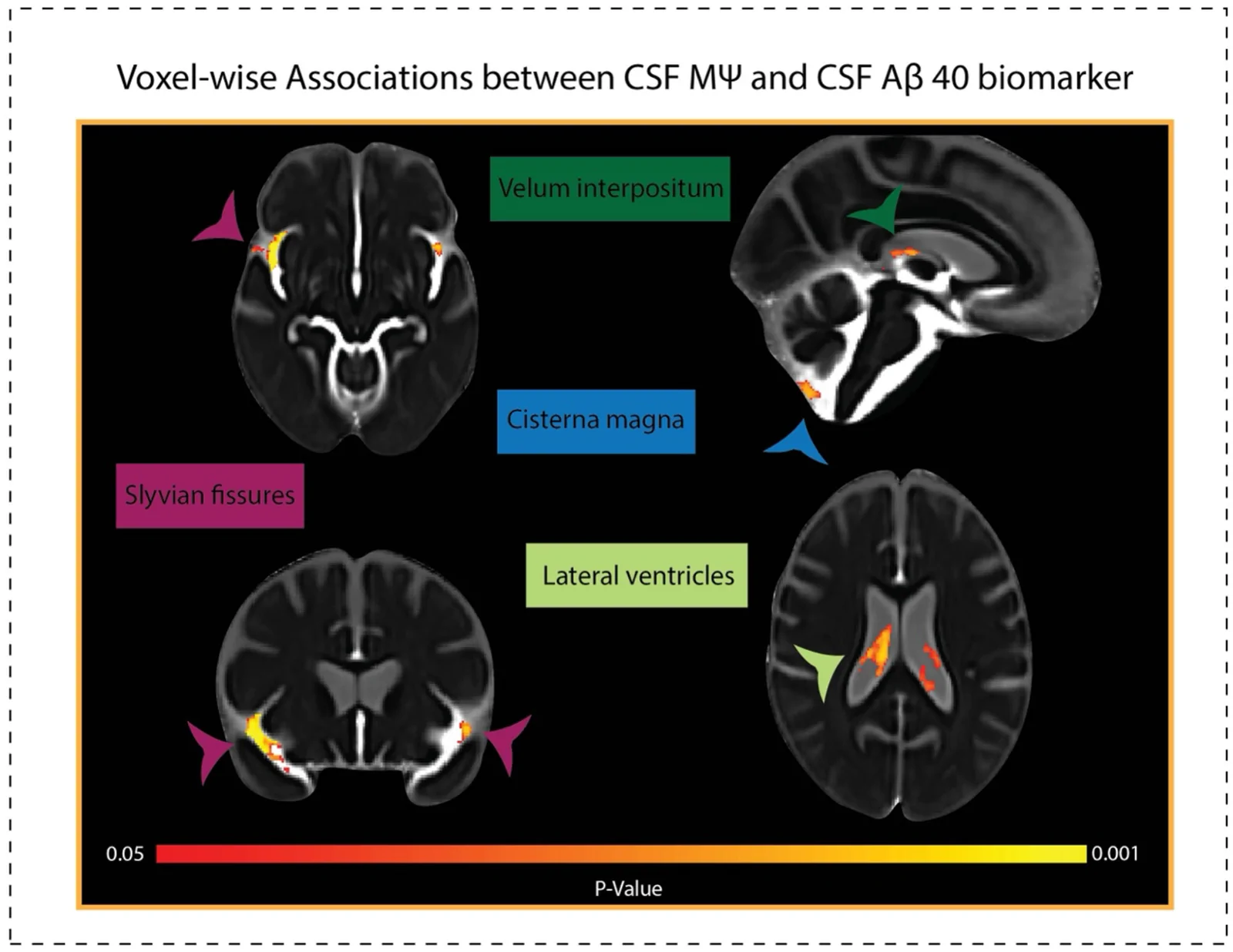
Multi-batch voxel-wise analysis. Voxel-wise analyses across all batches with whole-brain coverage (n=312), using the base model adjusted for age and sex, showed that higher harmonized CSF motility (MΨ) was associated with elevated CSF Aβ40 concentrations with the significant clusters mainly in the Sylvian fissures (left: *p*_*FWE*_ = 0.01; right: *p*_*FWE*_ = 0.001), lateral ventricles (left: *p*_*FWE*_ = 0.01; right: *p*_*FWE*_ = 0.009), velum interpositum cistern (*p*_*FWE*_ = 0.03) and cisterna magna (*p*_*FWE*_ = 0.01). Abbreviations: MΨ, mean pseudo-diffusivity; FWE, family-wise error

Following ROI-based analyses, voxel-wise analyses were performed to localize CSF motility-Aβ40 associations beyond predefined CWW regions. These analyses mirrored the ROI findings, with higher CSF Aβ40 was associated with higher CSF MΨ, with significant clusters in the Sylvian fissures (left: p_FWE_ = 0.01; right: p_FWE_ = 0.001), lateral ventricles (left: p_FWE_ = 0.01; right: p_FWE_ = 0.009), velum interpositum cistern (p_FWE_ = 0.03), and cisterna magna (p_FWE_ = 0.01; Figure 3). No voxel-wise associations were observed in the opposite direction for Aβ40, and no significant voxel-wise associations were observed for Aβ42 or the Aβ42/Aβ40 ratio.

### 3.3. Peri-Sylvian CSF motility is positively associated with plasma Aβ40

**Figure 4.**
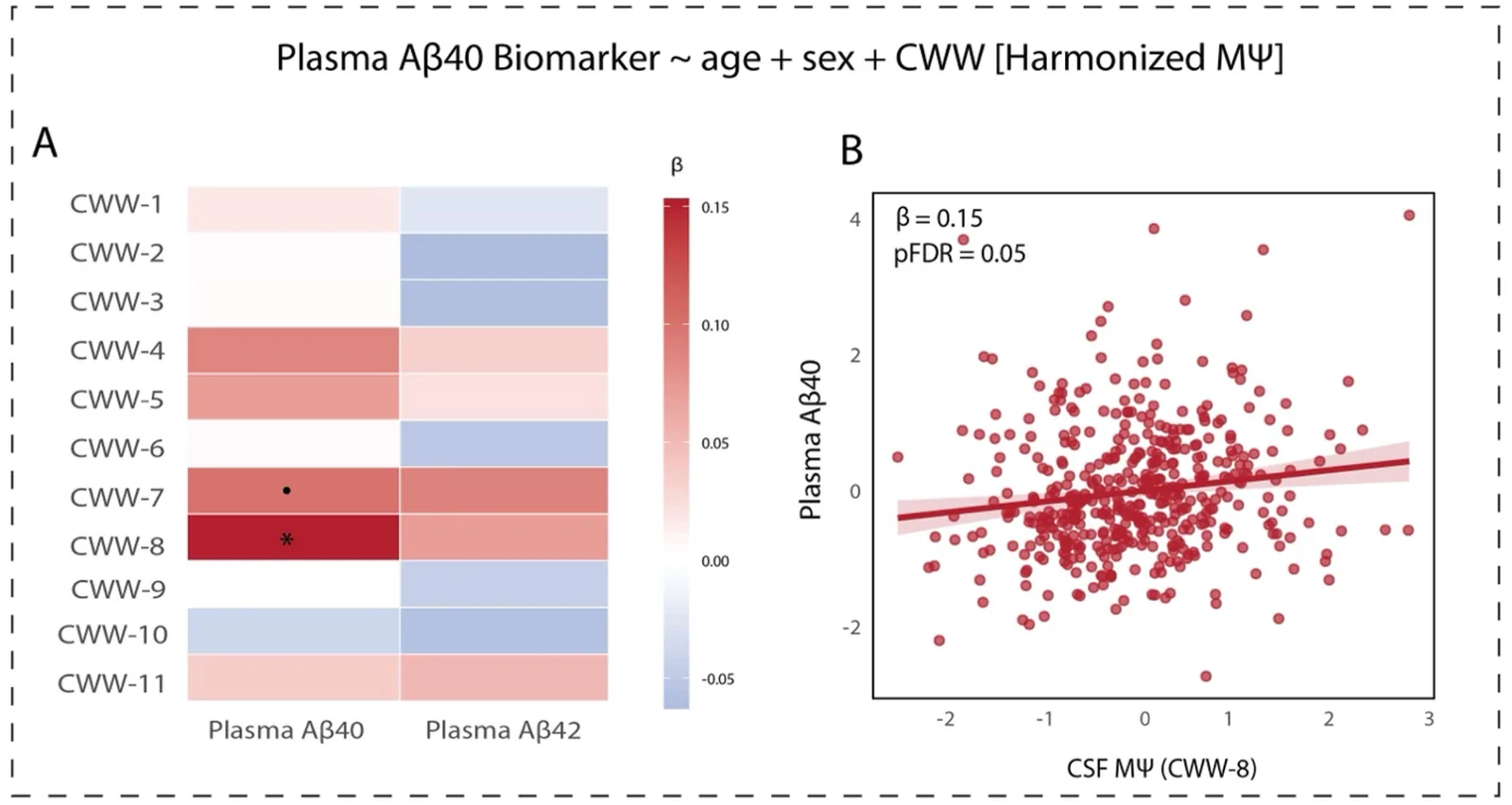
Effects of CSF mean pseudo-diffusivity on plasma biomarker levels across CSF Waterways. **(A)** ROI analyses across all batches (n=459) show the effects of harmonized CSF motility (MΨ) on plasma Aβ40 and Aβ42 concentrations across CWW regions, estimated using a base linear model adjusted for age and sex. Asterisks (*) indicate significant effects after FDR correction: p_FDR_ = 0.05 (*), and dots indicate nominal significance (p<0.05). **(B)** Higher CSF MΨ within the Sylvian fissure (CWW-8) was associated with increased levels of plasma Aβ40. Abbreviations: MΨ, mean pseudo-diffusivity; CWWs, CSF waterways; ROI, region-of-interest; BPF, brain parenchymal fraction; FDR, false discovery rate

Next, to assess the effect of CSF MΨ on plasma biomarkers concentration, ROI analyses were performed across all batches between harmonized CSF MΨ and plasma Aβ40 and Aβ42 levels in 459 participants. After adjusting for age, sex, increased CSF flow—particularly in the Sylvian fissure—was associated with higher plasma Aβ40 concentrations (CWW-8: β = 0.15, p_FDR_ = 0.05; Figure 4). This association persisted after additional adjustment for brain parenchymal fraction and CSF Aβ40 levels (n = 444, CWW-8: β = 0.13, p = 0.01). Exploratory interaction analyses indicated that CSF MΨ did not alter the relation between CSF and plasma Aβ40 (interaction term: CWW MΨ × CSF Aβ40). No significant association was observed for plasma Aβ42 in any of the CWW regions.

### 3.4. CSF motility shows no cross-sectional association with amyloid PET burden

**Figure 5.**
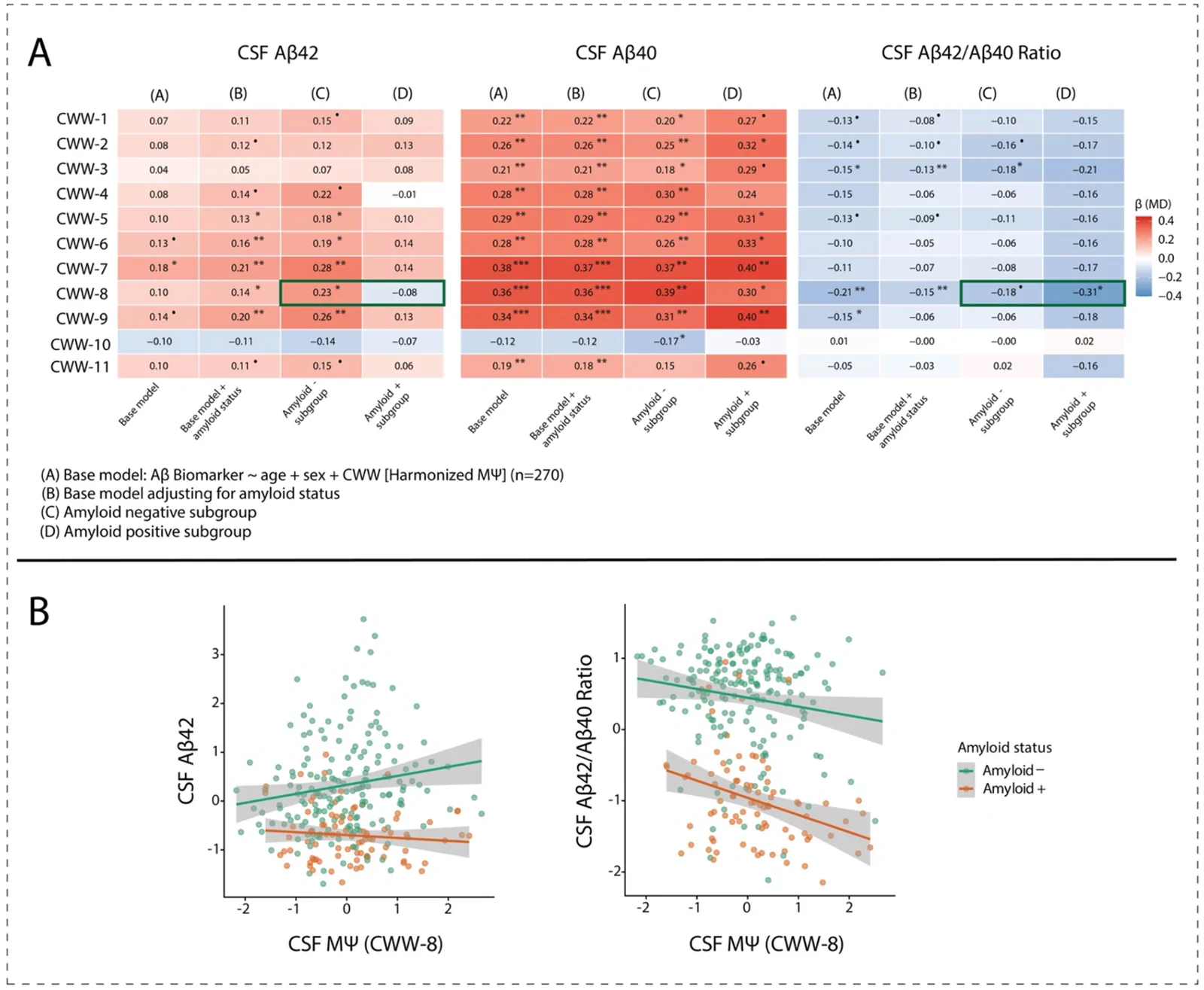
Effects of CSF mean pseudo-diffusivity on CSF Aβ biomarkers after accounting for amyloid pathology. **(A)** Heatmaps showing standardized β coefficients for associations between harmonized CSF motility (MΨ) and CSF Aβ biomarkers (CSF Aβ40, Aβ42, and the Aβ42/ Aβ40 ratio) across CWW regions. Models included: (A) the base model adjusted for age and sex; (B) the base model additionally adjusted for amyloid positivity; (C) the base model restricted to amyloid-negative participants; and (D) the base model restricted to amyloid-positive participants. Asterisks indicate significant effects after FDR correction: *p* < 0.05 (*), p < 0.01 (**), and p < 0.00001 (***), and dots indicate nominal significance (p<0.05). Green boxes indicate regions with nominally significant MΨ × amyloid status interaction effects. **(B)** Scatter plots show representative regions with significant interaction effects between CSF MΨ and amyloid status, estimated using the model: CSF Aβ biomarker ~ CWW [harmonized MΨ] × amyloid status + age + sex. Amyloid positivity was defined as PET Centiloid ≥18.41. Abbreviations: MΨ, mean pseudo-diffusivity; CWWs, CSF waterways; FDR, false discovery rate

We next evaluated the association between CSF flow and cerebral amyloid deposition using [18F] Florbetapir amyloid PET Centiloid values (n = 270). Across all batches, harmonized CSF MΨ showed no significant association with Centiloid values, indicating no cross-sectional relationship between regional CSF flow alterations and global amyloid burden. We then stratified participants by amyloid PET status. Associations between CSF MΨ and CSF Aβ40 were largely preserved in both amyloid-negative and amyloid-positive individuals, whereas inverse associations with the CSF Aβ42/Aβ40 ratio were more pronounced in amyloid-positive participants, particularly in peri-sylvian regions (CWW-8; amyloid negative: β = −0.18, unadjusted p = 0.03; amyloid positive: β = −0.31, p_FDR_ = 0.02). In contrast, positive associations between CSF motility and CSF Aβ42 were primarily observed in amyloid-negative participants and were attenuated in amyloid-positive individuals. The most prominent associations were observed in the peri-sylvian regions among amyloid-negative participants (CWW-7: β = 0.28, p_FDR_ = 0.001; CWW-8: β = 0.23, p_FDR_ = 0.02), whereas these associations were not significant in amyloid-positive individuals (CWW-7: β = 0.14, p = 0.2; CWW-8: β = −0.08, p = 0.5; Figure 5 and Supplementary Figure S2).

### 3.5 Regional CSF motility shows compartment-specific associations with Aβ kinetic parameters

**Figure 6.**
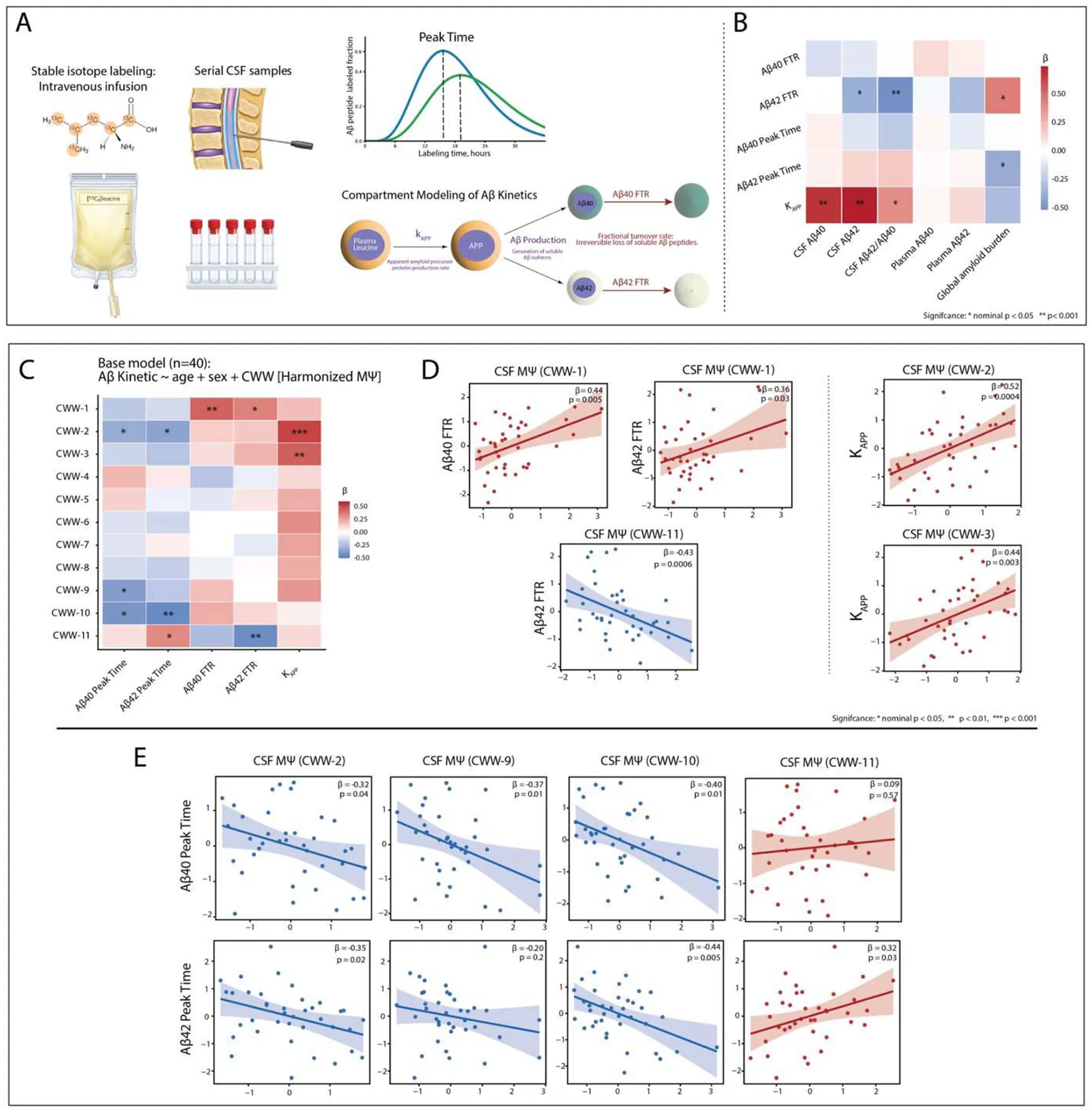
Effects of CSF mean pseudo-diffusivity on Aβ stable isotope labeling kinetics. **(A)** Schematic overview of the Aβ SILK protocol, including intravenous [^13^C_6_]-leucine infusion, serial CSF sampling via lumbar catheterization, and compartmental modeling to derive fractional turnover rate (FTR), peak time and APP production rate (K_APP_). **(B)** Heatmap illustrating the associations between Aβ kinetic parameters and CSF, plasma, and PET biomarkers adjusted for age and sex. **(C)** ROI analyses illustrating the association between harmonized CSF effective motility (MΨ) and Aβ kinetics across CSF Waterways in participants with available SILK data (n=40) estimated using the base model adjusted for age and sex. **(D-E)** Scatter plots visualize age- and sex-adjusted residualized and standardized associations between harmonized CSF MΨ and Aβ kinetic parameters (n=40). Abbreviations: MΨ, mean pseudo-diffusivity; CWWs, CSF waterways; ROI, region-of-interest; SILK, stable isotope labeling kinetics.

To directly assess the relationship between regional CSF effective motility and Aβ dynamics, we performed ROI-based analyses relating harmonized CSF MΨ to Aβ kinetic parameters in a subset of participants with MRI and SILK data acquired within six years of each other (n = 40; Figure 6A). To determine how SILK-derived Aβ kinetic measures relate to established markers of Aβ pathology, we first examined their associations with biofluid and PET biomarkers. Higher APP production rates (K_APP_) were strongly associated with higher CSF Aβ levels (CSF Aβ42: β = 0.74, p < 0.001; CSF Aβ40: β = 0.68, p < 0.001) and higher CSF Aβ42/Aβ40 ratio (β = 0.39, p = 0.02; n=39). In contrast, faster Aβ42 fractional turnover rates (FTR) were associated with lower CSF Aβ42 concentrations (β = −0.37, p = 0.02), a lower CSF Aβ42/Aβ40 ratio (β = −0.51, p < 0.001; n = 39), and greater global amyloid PET burden (β = −0.44, p = 0.01; n = 34). Additionally higher global amyloid PET burden was found to be linked with earlier peak time for Aβ42 (β = −0.38, p = 0.03; n=33; Figure 6B). Aβ40 kinetic measures, including FTR and peak time, showed no significant associations with Aβ-related biomarkers, and plasma Aβ levels were not associated with CSF SILK-derived kinetic parameters.

Higher CWW-1 MΨ was associated with faster Aβ40 FTR (β = 0.44, p = 0.005) and faster Aβ42 FTR (β = 0.36, p = 0.03), whereas higher extra-axial CWW-11 MΨ was associated with slower Aβ42 FTR (β = −0.43, p = 0.006; Figure 6C and 7D). Higher CSF MΨ was associated with earlier peak times for both Aβ40 and Aβ42 within third and fourth ventricles (CWW-10; Aβ40: β = −0.4, p = 0.01; Aβ42: β = −0.44, p = 0.005) and the cranio-cervical junction (CWW-2; Aβ40: β = −0.32, p = 0.04; Aβ42: β = −0.35, p = 0.02). For Aβ40, CSF MΨ within the supracerebellar and velum interpositum cisterns (CWW-9) was additionally associated with earlier peak times (β = −0.37, p = 0.01). In contrast, within extra-axial CSF spaces (CWW-11), higher CSF MΨ was associated with a delayed peak time for Aβ42 (β = 0.32, p = 0.03; Figure 6C and 7E). Furthermore, higher CSF motility within cranio-cervical junction (CWW-2; β = 0.52, p < 0.001) and superior premedullary cistern (CWW-3; β = 0.44, p = 0.003) was highly correlated with K_APP_ (Figure 6C and 7D). Adjustment for amyloid deposition did not substantially alter the associations between CWW MΨ and peak times or K_APP_. However, adjustment attenuated the association between extra-axial CWW-11 MΨ and Aβ42 FTR (Supplementary Figure S3).

## 4. Discussion

The association between CSF motility (MΨ) and CSF Aβ40 concentrations was robust across imaging parameters and analytic approaches. ROI-based analyses using the CWW atlas demonstrated that higher effective CSF motility across multiple ventricular and extra-axial CSF compartments was associated with higher CSF Aβ40 concentrations across multiple imaging batches with differing acquisition parameters. These associations were preserved after adjustment for brain atrophy, including lateral ventricular volume. Voxel-wise analyses closely mirrored these regional findings, demonstrating convergent and spatially consistent associations. Overall, regional CSF motility accounted for approximately 18% of the variance in CSF Aβ40 concentrations.

Associations between CSF motility and CSF Aβ42 were weaker and more anatomically restricted to the Sylvian and interhemispheric fissures and the extra-axial subarachnoid space. Moreover, CSF MΨ was not associated with global amyloid burden on PET imaging, indicating that CSF flow at the time of measurement is not related to contemporaneous parenchymal amyloid deposition, while leaving open the possibility that altered CSF dynamics may influence subsequent amyloid accumulation. Associations between CSF motility and CSF Aβ42 were stronger among amyloid-negative individuals, suggesting that once substantial parenchymal amyloid deposition is established, plaque sequestration may attenuate the relationship between CSF motility and soluble CSF Aβ42 levels. These observations may also represent distinct biochemical and pathophysiological profiles of Aβ40 and Aβ42: Aβ42 differs from Aβ40 by two additional C-terminal residues that confer a more rigid, aggregation-prone conformation, reducing its water solubility and increasing its propensity for parenchymal plaque formation in AD^55,56^.

Higher peri-sylvian CSF motility (CWW-8) was also associated with higher plasma Aβ40 levels. This association situates peri-arterial CSF dynamics within a broader vascular context, as elevated plasma Aβ40 has been linked to subclinical atherosclerosis, cardiovascular disease, and intracranial small-vessel disease markers, including white matter hyperintensities, lacunes, and cerebral microbleeds^57–61^. Given that vascular amyloid deposition in CAA is enriched in Aβ40, this finding may also be relevant to amyloid-related cerebrovascular dysfunction, consistent with prior reports associating higher plasma Aβ40 with CAA^59,61^ with recent evidence of increased peri-arterial CSF motility in patients with CAA^62^. Notably, the peri-sylvian CSF compartment surrounds the middle cerebral arteries and shows age-related increases in CSF MΨ. Higher peri-sylvian CSF motility may therefore reflect increased transmission of arterial pulsatility into adjacent CSF spaces as vascular stiffness increases and pulsatile dampening declines with age^63^. Together, these observations raise the possibility that the association between peri-sylvian CSF motility and plasma Aβ40 reflects a vascular-pulsatility phenotype linking arterial aging, altered periarterial CSF dynamics, circulating Aβ40, and potentially CAA.

The regional pattern of associations between CSF motility and CSF Aβ concentrations observed in this study may reflect distinct underlying mechanisms. We therefore leveraged SILK technology to uniquely quantify Aβ production, turnover, and clearance dynamics in human central nervous system, *in vivo*^25,27,28^. SILK-based kinetic analyses provide mechanistic insight into the kinetic basis of associations between regional CSF motility and CSF Aβ levels. Among the SILK-derived measures, the apparent amyloid precursor protein production rate (K_APP_) showed the strongest association with CSF Aβ levels. Notably, K_APP_ was strongly associated with CSF motility in the lower basilar cisterns and craniocervical junction (CWW-2 and CWW-3). CSF composition varies across compartments, with established craniospinal concentration gradients^64,65^. Given that CSF Aβ40 concentrations and apparent K_APP_ were both derived from lumbar CSF, their values likely depend on intracranial-spinal CSF exchange. Their concordant associations with higher craniocervical junction CSF effective motility may therefore reflect more efficient mixing and Aβ transport across these compartments. Higher CSF effective motility at both the craniocervical junction (CWW-2) and along the transventricular pathway (CWW-10) was associated with earlier peak time, may reflect more rapid mixing between CSF compartments. The distinct inverse association between transventricular CSF motility and lumbar CSF Aβ40 and Aβ42 levels may reflect greater mixing with ventricular CSF, where Aβ concentrations have been reported to be lower than in lumbar CSF^66^. Overall, these findings suggest that regional CSF effective motility may shape lumbar Aβ concentrations and kinetics by modulating mixing and exchange among spinal, ventricular, and extraventricular intracranial CSF compartments.

Higher effective motility within the lateral ventricles (CWW-1) was associated with faster Aβ40 and Aβ42 turnover (FTR). Although the underlying mechanism remains uncertain, one possible explanation is that intraventricular CSF motility reflects CSF production dynamics, such that enhanced CSF production is accompanied by more rapid Aβ clearance and turnover. In contrast to other CSF compartments, higher extra-axial CSF motility (CWW-11) was associated with delayed Aβ42 peak timing and reduced FTR. The interpretation of this Aβ42-specific reversal remains uncertain and may indicate that increased motility in extra-axial spaces reflects processes distinct from bulk transport efficiency. Importantly, SILK-derived kinetic measures did not fully account for the regional associations between CSF effective motility and Aβ. For example, peri-sylvian CSF motility showed the strongest association with CSF Aβ40 concentrations but was not associated with the measured SILK kinetic parameters, suggesting that additional mechanisms, potentially operating over longer timescales than those captured by SILK, may contribute to regional CSF motility-Aβ relationships.

Prior human studies have primarily relied on phase-contrast MRI to evaluate CSF flow, an approach inherently limited by its restricted field of view. To date, few studies have directly examined relationships between regional CSF flow and amyloid burden or amyloid concentrations in CSF or plasma. In one study, aqueductal CSF flow measured with phase-contrast MRI was not associated with cerebral amyloid deposition^67^, consistent with our findings. In another study, MRI markers of normal-pressure hydrocephalus were associated with lower CSF Aβ42 in the absence of parenchymal amyloid deposition. In that context, these findings suggest that normal pressure hydrocephalus-related disturbances in CSF circulation and turnover may influence CSF amyloid concentrations and apparent CSF-based clearance without necessarily affecting parenchymal amyloid deposition^68^.

There are several limitations that warrant consideration. First, our cohort consisted primarily of older adults without dementia (85.88% with a CDR of 0) with the overall amyloid positivity of 17%. As such, the findings may not directly generalize to individuals with established CAA; evaluating CSF flow alterations in a well-characterized CAA cohort will be an important next step. Second, our analysis relied on CSF MΨ derived from low-b dMRI as a surrogate of effective CSF motility. Although this metric captures spatial patterns of overall CSF motility, it does not encompass the full multidimensional complexity of CSF flow. Emerging approaches such as dynamic low-b dMRI to assess variation across the cardiac cycle^19^, multi-directional low-b acquisitions to characterize flow directionality^21^, and 4D phase-contrast MRI^69^ for quantitative velocity-resolved CSF flow mapping will be essential for a more comprehensive characterization of CSF dynamics. Third, our analyses of amyloid PET were cross-sectional, limiting inference about how CSF motility influences amyloid accumulation or deposition over time; longitudinal imaging will be required to determine whether altered CSF dynamics modulate the trajectory of amyloid deposition.

In conclusion, our findings identify CSF motility as an important physiological determinant of soluble Aβ levels and kinetics, with stronger cross-sectional associations for Aβ40 than Aβ42. In contrast, CSF motility showed no strong association with parenchymal amyloid deposition, indicating that CSF flow primarily modulates soluble Aβ dynamics. Our findings suggest that CSF motility shapes CSF-mediated Aβ clearance through a multi-step, compartmental process. In this framework, extraventricular intracranial CSF spaces may be more closely coupled to interstitial fluid and APP-derived Aβ production, whereas subsequent Aβ distribution and removal through the subarachnoid space may depend on CSF production, turnover, and exchange across CSF compartments. Clinically, these findings suggest that CSF motility metrics derived from low-b dMRI; readily obtainable from standard diffusion sequences already included in baseline and longitudinal MRI protocols for amyloid-related imaging abnormalities (ARIA) monitoring, could provide a scalable, noninvasive physiological marker to stratify cerebrovascular amyloid clearance efficiency and potentially identify individuals at higher risk for ARIA; a key treatment-limiting complication of anti-amyloid therapies or enable early detection of CAA-related vulnerability^70,71^. Beyond Aβ, CSF circulation may also contribute to inter-individual variability in other CSF and brain-derived plasma biomarkers. Understanding these dynamics could provide insights into the pathophysiology of multiple neurodegenerative diseases and inform the development of fluid-based diagnostic biomarkers.

## Supplementary Material

1

## Figures and Tables

**Table 1.** Demographic, clinical, and imaging characteristics of the low-b dMRI batches.

Batch (n)	Age (Y, mean ± SD)	Sex (Female/Male)	CDR (n)	Amyloid positivity (%)	APOE ε4 carriers (%)
mMR 1 (n=101)	67.9 ± 7.5	60/41	0:89; 0.5:12; 1:0	26.7	37.6
mMR 2 (n=43)	69.7 ± 8.5	28/15	0:34; 0.5: 5; 1:4	39.5	39.5
mMR 3 (n=45)	71.3 ± 7.4	20/25	0: 39; 0.5: 6; 1:0	31.1	31.1
mMR 4 (n=93)	70.1 ± 8.1	45/48	0: 82; 0.5: 6; 1:4	29.0	37.6
Vida 5 (n=73)	69.7 ± 8.8	36/37	0: 64; 0.5:7: 1:2	-	41.1
Vida 6 (n=26)	66.9 ± 10.8	13/13	0: 25; 0.5: 1; 1:0	-	30.7
TrioTim (n=121)	68.8 ± 8.7	72/49	0:98; 0.5:19; 1:2	-	37.1
