# Supplementary material for: Regional Cerebrospinal Fluid Motility as a Key Determinant of Soluble Amyloid-β Levels and Kinetics
Source: Res Sq. 2026 Aug 3:rs.3.rs-10530209. Preprint. [Version 1] doi: 10.21203/rs.3.rs-10530209/v1 (PMC13484840; doi:10.21203/rs.3.rs-10530209/v1)
Supplement: Supplementary file 1 [file NIHPPRS10530209v1-supplement-1.pdf]

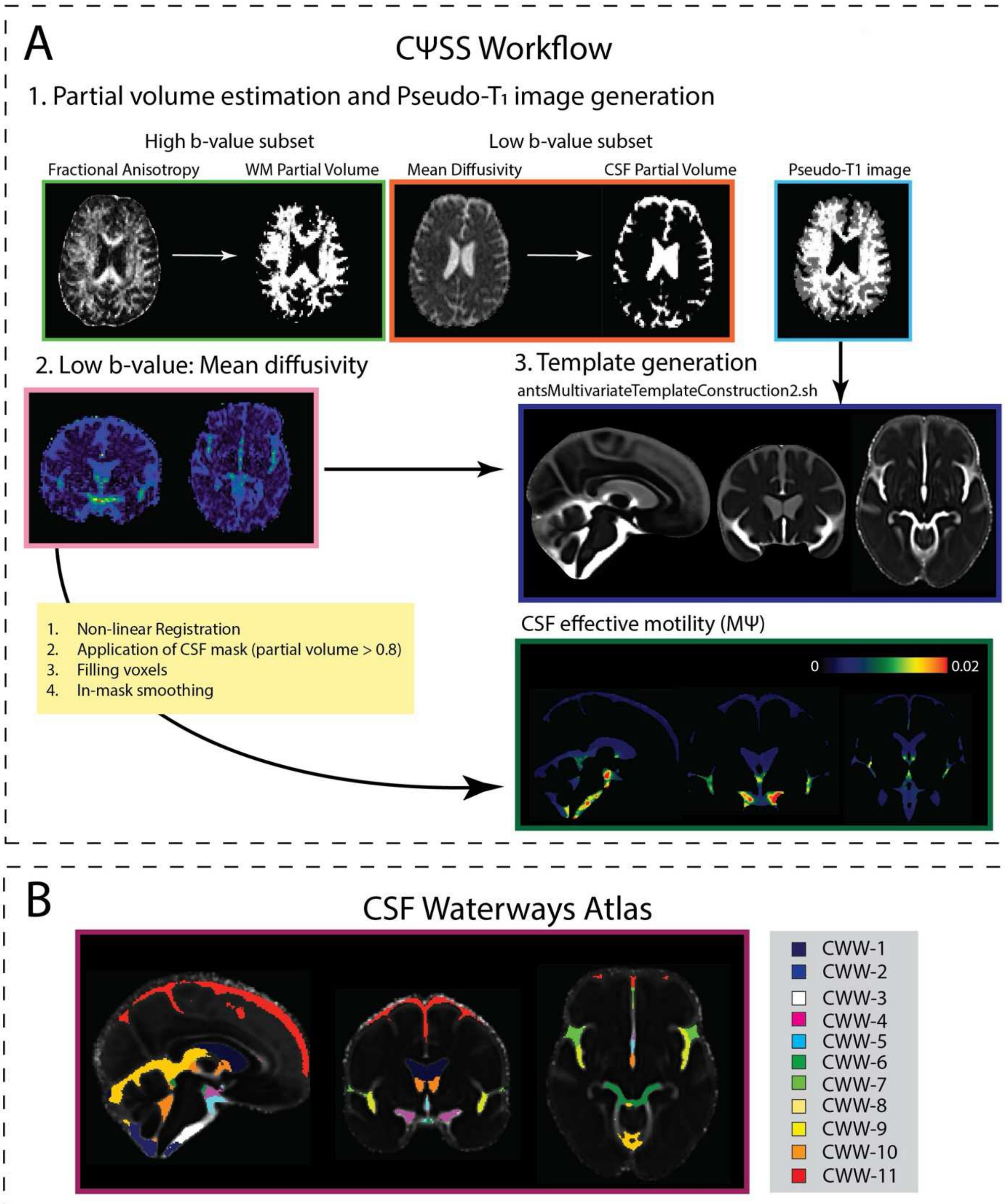

Supplementary Figure S1. Overview of CSF Pseudo-diffusion Spatial Statistics (CΨSS) and CSF Waterways (CWW) atlas. (A) For CΨSS, the following steps were performed: (1) multi-b-value diffusion MRI was divided into low b-value (b=0-450 s/mm^2) and high b-value (b≥ 500 s/mm^2) subsets. The Diffusion tensor model was fitted to the high b-value subset to

obtain FA and to the low b-value subset to obtain mean diffusivity. These two were then fed into Atropos (part of ANTs) to generate partial volume maps and pseudo-T1-weighted images. (2) Low b-value subset (b=0-450 s/mm^2) were used to generate low b-value MΨ maps, measuring effective CSF motility. (3) MΨ and pseudo-T1 images were utilized to create batch-specific templates using *antsMultivariateTemplateConstruction2.sh* (ANTs). CSF probability maps were subsequently registered to the common template space. To account for partial volume effects, the final CSF mask was generated by retaining voxels with CSF fraction > 0.8 in more than 75% subjects. Voxels with a CSF fraction < 0.8 were filled using surrounding voxel average. Spatial smoothing (2 mm FWHM) was applied within the CSF mask using 3dBlurInMask (part of AFNI).

Abbreviations: CSF, cerebrospinal fluid; FA, fractional anisotropy; MΨ, mean pseudo-diffusivity; FWHM, full width half maximum.

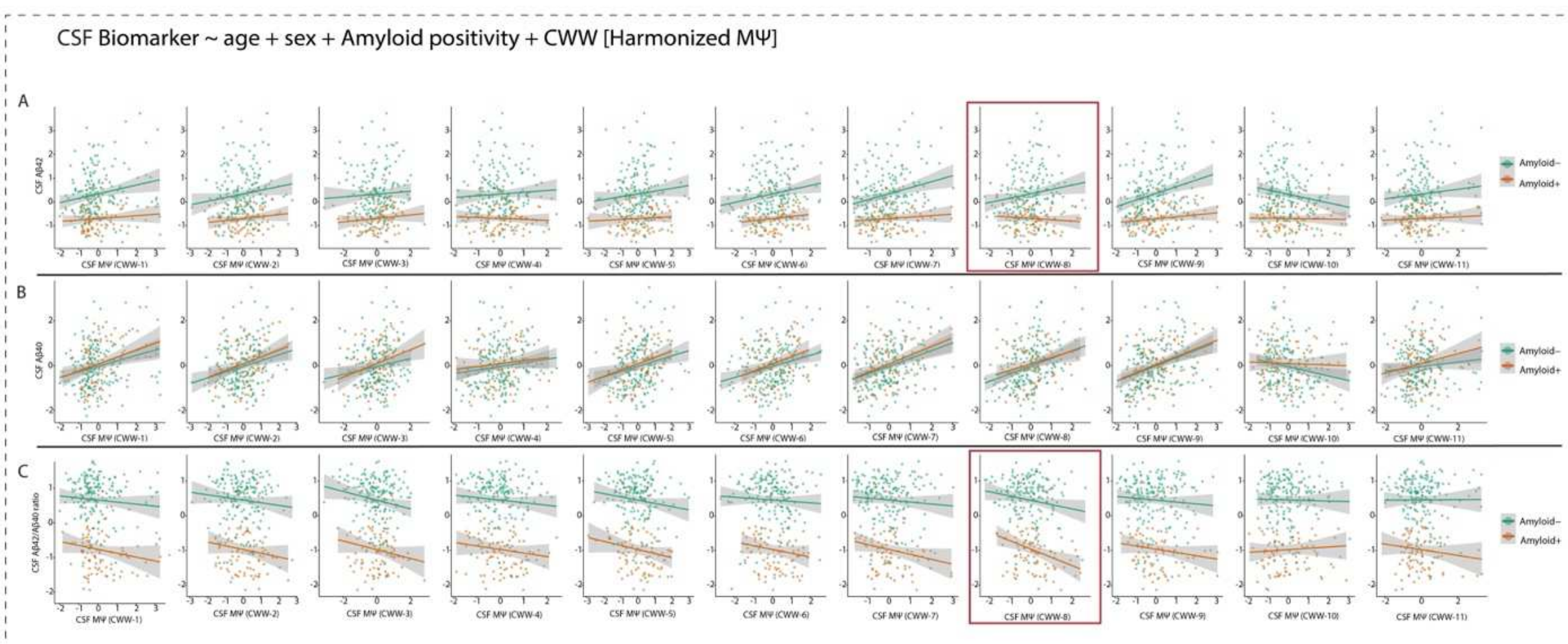

Supplementary Figure S2. Stratified associations between harmonized CSF mean pseudo-diffusivity and CSF Aβ biomarkers based on amyloid status. Scatter plots show associations between age and sex adjusted CSF motility (MΨ) and CSF Aβ biomarkers (CSF

Aβ40, Aβ42, and the Aβ42/ Aβ40 ratio) across CWW regions, stratified by amyloid status. Lines represent subgroup-specific linear fits with shaded 95% confidence intervals. Red boxes indicate regions with nominally significant MΨ × amyloid status interaction effects.

Abbreviations: MΨ, mean pseudo-diffusivity; CWWs, CSF waterways

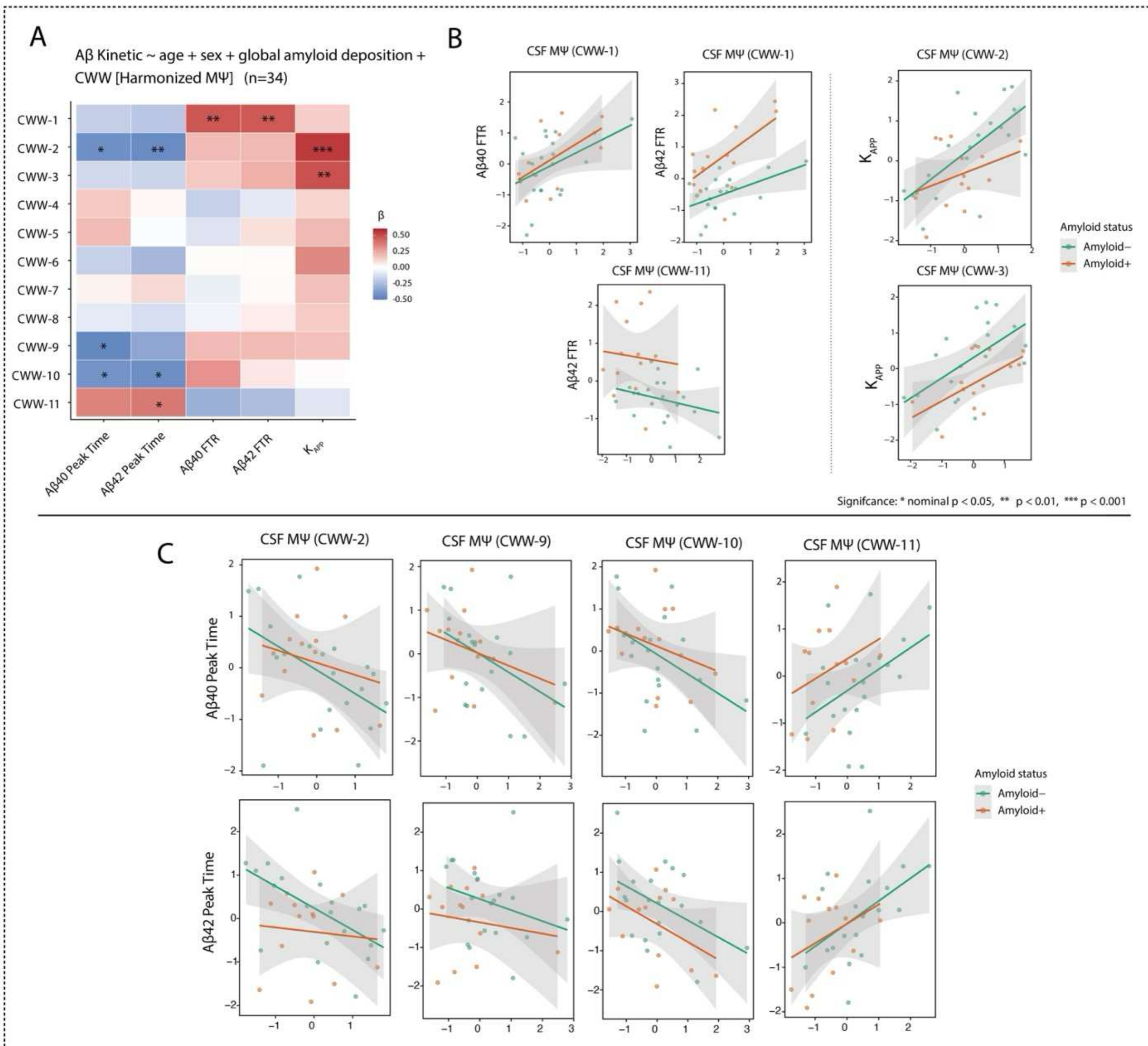

Supplementary Figure S3. Effects of CSF mean pseudo-diffusivity on Aβ stable isotope labeling kinetics after adjustment for global amyloid burden. (A) ROI analyses illustrating

the association between harmonized CSF effective motility (MΨ) and Aβ kinetics across CSF Waterways in participants with available amyloid PiB PET data (n = 34), estimated using linear models adjusted for age, sex, and global amyloid burden. (B-C) Scatter plots visualize age- and sex-adjusted residualized and standardized associations between harmonized CSF MΨ and Aβ kinetic parameters stratified by amyloid status in participants with available amyloid PiB PET data (n = 34).

Abbreviations: MΨ, mean pseudo-diffusivity; CWWs, CSF waterways; ROI, region-of-interest; SILK, stable isotope labeling kinetics; PiB, Pittsburgh compound B.

Scanner (3T Siemens)	Batch	TR/TE (ms)	Volumes (n)	Number of low b-values	b-value (s/mm2)	Slice number
Biograph mMR	mMR 1	9500/93	82	27	0-2000	60
	mMR 2	10200/92	82	27	0-2000	60
	mMR 3	10300/86	26	9	0-1400	80
	mMR 4	13300/99	26	9	0-1400	80
Vida	Vida 5	5800/79	69	29	0-2000	80
	Vida 6	9600/79	69	29	0-2000	80
TrioTim	TrioTim 7	9900/102	26	12	0-1000	60

Supplementary Table S1 *Acquisition parameters of low-b dMRI across 3T Siemens MRI scanners.*

Batch (n)	CSF Aβ42	CSF Aβ40	CSF Aβ42/Aβ40	Plasma Aβ40	Plasma Aβ42	Aβ PET
mMR 1 (n=101)	915.9 ± 399.3	12096.9 ± 3740.1	0.08 ± 0.02	448.9 ± 67.7	44.6 ± 7.5	12.0 ± 31.1
mMR 2 (n=43)	758.7 ± 286.2	11291.8 ± 3395.4	0.07 ± 0.02	460.4 ± 71.2	46.6 ± 7.3	30.0 ± 46.1
mMR 3 (n=45)	823.7 ± 253.6	11719.1 ± 2729.3	0.07 ± 0.02	441.7 ± 73.8	44.3 ± 6.7	12.8 ± 36.1
mMR 4 (n=93)	733.3 ± 309.3	10468.2 ± 3302.5	0.07 ± 0.02	419.6 ± 75.1	41.8 ± 6.4	14.1 ± 35.3
Vida 5 (n=73)	832.9 ± 381.2	11662.0 ± 3450.8	0.07 ± 0.02	434.0 ± 52.3	43.2 ± 4.9	No PET data available
Vida 6 (n=26)	888.5 ± 354.3	11060.2 ± 3105.9	0.08 ± 0.02	438.6 ± 66.1	44.9 ± 7.8	No PET data available
TrioTim (n=121)	789.8 ± 361.1	11279.1 ± 3549.1	0.07 ± 0.02	425.8 ± 69.7	43.2 ± 7.6	No PET data available

Supplementary Table S2. *Summary of Amyloid Beta biomarkers across scanner batches.*

Values are reported as mean ± SD. CSF and plasma concentrations are in pg/mL. Aβ PET values are AV-45 derived Centiloid measures of global amyloid burden.
